# The PTI to ETI Continuum in *Phytophthora*-Plant Interactions

**DOI:** 10.3389/fpls.2020.593905

**Published:** 2020-12-17

**Authors:** Zunaira Afzal Naveed, Xiangying Wei, Jianjun Chen, Hira Mubeen, Gul Shad Ali

**Affiliations:** ^1^Department of Plant Pathology, Institute of Food and Agriculture Sciences, University of Florida, Gainesville, FL, United States; ^2^Mid-Florida Research and Education Center, Institute of Food and Agriculture Sciences, University of Florida, Apopka, FL, United States; ^3^Institute of Oceanography, Minjiang University, Fuzhou, China; ^4^Departement of Biotechnology, University of Central Punjab, Lahore, Pakistan; ^5^EukaryoTech LLC, Apopka, FL, United States

**Keywords:** *Phytophthora*, plant defense, plant immunity, zigzag model, PAMPS, effectors, RXLR, susceptibility genes

## Abstract

*Phytophthora* species are notorious pathogens of several economically important crop plants. Several general elicitors, commonly referred to as Pathogen-Associated Molecular Patterns (PAMPs), from *Phytophthora* spp. have been identified that are recognized by the plant receptors to trigger induced defense responses in a process termed PAMP-triggered Immunity (PTI). Adapted *Phytophthora* pathogens have evolved multiple strategies to evade PTI. They can either modify or suppress their elicitors to avoid recognition by host and modulate host defense responses by deploying hundreds of effectors, which suppress host defense and physiological processes by modulating components involved in calcium and MAPK signaling, alternative splicing, RNA interference, vesicle trafficking, cell-to-cell trafficking, proteolysis and phytohormone signaling pathways. In incompatible interactions, resistant host plants perceive effector-induced modulations through resistance proteins and activate downstream components of defense responses in a quicker and more robust manner called effector-triggered-immunity (ETI). When pathogens overcome PTI—usually through effectors in the absence of R proteins—effectors-triggered susceptibility (ETS) ensues. Qualitatively, many of the downstream defense responses overlap between PTI and ETI. In general, these multiple phases of *Phytophthora-*plant interactions follow the PTI-ETS-ETI paradigm, initially proposed in the zigzag model of plant immunity. However, based on several examples, in *Phytophthora*-plant interactions, boundaries between these phases are not distinct but are rather blended pointing to a PTI-ETI continuum.

## Introduction

Oomycetes are a unique class of eukaryotes classified in the kingdom Protoctista along with algae, diatoms, and other planktons under the sub kingdom Stramenopila (Kamoun et al., 2015). Morphologically they are quite similar to filamentous fungi but physiologically, genetically, and biochemically they are very different from fungi. Phytopathogenic oomycetes from the genus *Phytophthora* cause destructive diseases in many economically important crops and forest ecosystems (Hansen, 2015). Control strategies for Phytophthora diseases are very limited (Attard et al., 2014). Despite their differences, fungi and oomycetes use quite similar strategies to infect and colonize their hosts, but many chemicals that target sterol synthesis and chitin in fungal cell walls, which are absent from oomycetes, do not control diseases caused by oomycetes (Latijnhouwers et al., 2003; Attard et al., 2014). Deployment of host genetic resistance is considered the most effective, eco-friendly and cost-effective management strategy. To develop sustainable resistance against oomycetes, a thorough understanding of the molecular basis of *Phytophthora*-plant interactions is very important (Anderson et al., 2010).

Plant defense comprises both constitutive preformed and induced defense responses that offers several layers of protection against pathogen invasion (Anderson et al., 2010; Doughari, 2015). Constitutive defense consisted of physical or chemical barriers that restrict the attachment and entry of the most microbes into the plants (Osbourn, 1996; Chassot et al., 2008; Underwood, 2012). According to the zigzag model of plant-pathogen interaction, induced defense consists of two layers, the first one is known as pathogen associated molecular pattern (PAMP) triggered immunity (PTI). In PTI, conserved molecules or structures of pathogens are perceived by plant pattern recognition receptors (PRRs), followed by activation of defense responses. To circumvent PTI, pathogens deliver effector proteins inside host cells, where they interfere with defense responses. Plants perceive effectors through resistance (*R)* genes and activate a more robust and faster defense response, termed as effector-triggered immunity (ETI). When one or more pathogen effectors suppress PTI, pathogens successfully infect susceptible hosts and in the absence of effective R proteins, ETI is overcome, eventually leading to effector triggered susceptibility (ETS). Multiple shifts between ETS and ETI occur because of coevolution of effectors in pathogens and corresponding *R* genes in plant hosts (Jones and Dangl, 2006). Although the real-world applicability of this model has been challenged (Thomma et al., 2011; Pritchard and Birch, 2014), this is so far considered the most concise model of plant immunity for pedagogical purposes. The popularity of zigzag model could be attributed to the fact that it elegantly contrives the three principal outcomes of plant-pathogen interactions: the non-race-specific elicitor-induced defense (PTI), the race or cultivar-specific gene-for-gene resistance (ETI), and race-specific pathogen virulence and host susceptibility (ETS).

In the zigzag model, PTI and ETI are viewed as two separate sequential branches of plant immunity (Jones and Dangl, 2006). However, there are several examples where effectors fulfill the criteria to be designated as PAMPs (Thomma et al., 2011) and PAMPs as effectors. Moreover, in the zigzag model the term *R* gene was exclusively reserved for intracellular receptors harboring nucleotide binding (NB) and leucine rich repeat (LRR) domains. However, in the original gene-for gene resistance concept, Flor described both transmembrane as well as intracellular pathogen receptors as R proteins (Thomma et al., 2011). Accordingly, PRRs, at least some of them, have been classified as a subclass of *R* genes (Sanseverino et al., 2012). Based on several plant-pathogen interaction examples, Thomma et al., proposed a continuum between PTI and ETI, in which the strict boundaries of the zigzag model are blurred (Thomma et al., 2011). In this review, we have discussed the current state of *Phytophthora-*plant interactions in terms of zigzag model as well as deviations from this model. These discussions suggest that the PTI-ETI continuum concept also applies to *Phytophthora-*plant interactions. The application of current knowledge about *Phytophthora-*plant interactions to develop broad-spectrum long-lasting resistance in plants against *Phytophthora* spp. has also been discussed.

## Pamp Triggered Immunity (PTI)

Plants recognize *Phytophthora* spp. by sensing a wide variety of elicitors (Table 1). The term “elicitor” was initially used to refer to both the exogenous and endogenous signaling molecules that can induce any defense response in plants (Eder and Cosio, 1994; Hahn, 1996). Exogenous elicitors could be either the components of pathogen's cell wall or membranes, released upon action of host enzymes or secreted by the pathogen during host-pathogen interaction to subvert the host defense and/or facilitate nutrient acquisition (Raaymakers and Van Den Ackerveken, 2016). Whereas, the endogenous elicitors were referred to components that originated from host plants, usually resulting from damage caused by the action of pathogen enzymes (Eder and Cosio, 1994). In the zigzag model, the terms exogenous and endogenous elicitors were replaced by PAMPs and DAMPs (Damage Associated Molecular Patterns), respectively and the induced defense upon recognition of either PAMP or DAMP was termed as PTI. PAMPs fall into a broad spectrum of chemistries ranging from carbohydrates to proteins as is discussed below.

**Table 1:** *Phytophthora* spp. elicitors and associated PTI components.

**Name**	**Pathogen groups carrying PAMP**	**Chemical nature**	**Cognate PRR**	**PAMP perception model**	**Signaling**	**References**
Elicitins	Unique to oomycete genera; *Phytophthora* and *Pythium*	Protein	ELR (Reported for Cryptogein, INF1 and ParA1)	BAK1/SERK3 dependent (Reported for INF1 and ParA1)	SA (Cryptogein) JA and ET (INF1) Calcium and MAPK (Cryptogein and INF1)	Kamoun et al., 1998; Lebrun-Garcia et al., 1998; Kawamura et al., 2009; Amelot et al., 2011; Du Y. et al., 2015; Peng et al., 2015; Derevnina et al., 2016
OPEL	Oomycetes	Protein	Unknown	Unknown	SA	Chang et al., 2015
Pep-13	*Phytophthora* spp.	Protein/peptide	Unknown	BAK1/SERK3 independent	SA and JA Calcium and MAPK	Nürnberger et al., 1994; Blume et al., 2000; Halim et al., 2009; Wang H. et al., 2019
β-glucans	Oomycetes and fungi	Carbohydrate	Unknown	Unknown	SA	Kopp et al., 1989; Klarzynski et al., 2000; Fesel and Zuccaro, 2016
Eicosapolyenoic acids (EPs)	Oomycetes, primitive fungi and nematodes	Lipids	Unknown	Unknown	JA	Preisig and Kuć, 1985; Savchenko et al., 2010
**Elicitors with dual, PAMP and effector status**						
XEG1-GH12	Fungi and oomycetes	Protein-CWDEs	Unknown	BAK1/SERK3 dependent	Unknown	Ma et al., 2015, 2017
CBEL-CBM1	Fungi and oomycetes	Protein-CWDEs	Unknown	BAK1/SERK3 dependent	SA, JA, and ET	Khatib et al., 2004; Larroque et al., 2013
nlp20-NLPs	Bacteria, fungi and oomycetes	Protein	RLP23	BAK1/SERK3 dependent	SA	Böhm et al., 2014; Albert et al., 2015

### Proteinaceous PAMPs of *Phytophthora* spp.

Elicitins are extracellular structurally conserved cysteine-rich proteins secreted by species in the genus *Pythium* and *Phytophthora*. They do not display sequence similarity to plant proteins, and thus are recognized as non-self-molecules by the host plants leading to the induction of an array of defense responses (Derevnina et al., 2016). Elicitins are reported to bind sterols and lipids. Sterols are certain types of steroid alcohols, which are essential structural and functional components of eukaryotes. *Pythium* and *Phytophthora* lack sterol synthesis ability, and, therefore, rely on their host's sterols. They have adapted efficient mechanisms of sterol scavenging from the host cell membranes, possibly through elicitins (Mikes et al., 1998). Elicitins are designated as oomycetes PAMPs that can induce PTI. In non-host plants, pathogens are not capable of overcoming this elicitin-induced PTI, rendering pathogens as non-adapted; these interactions are called non-host/non-pathogen interactions.

Non-host resistance has long been studied using the tobacco-*Phytophthora* model because most of the *Phytophthora* spp. are non-adapted pathogens of tobacco. For example, cryptogein isolated from *P. cryptogea* is one of the earliest identified sterol-binding elicitins, which has been extensively used to explore defense mechanisms underlying non-host resistance. The sterol-cryptogein complex is recognized by the tobacco PRRs and triggers a strong defense response (Lochman et al., 2005), likely making *P. cryptogea*, a non-adapted pathogen of tobacco (Wendehenne et al., 2002). A number of elicitins from different *Phytophthora* spp. have been identified and they are being used to identify host factors that can offer broad spectrum non-race specific resistance to diverse phytopathogens (Du J. et al., 2015). These include INF1 from *P. infestans*, ParA1 from *P. parasitica*, CAP1 from *P. capsici*, PAL1 from *P. palmivora* and Quercinin from *P. quercina* (Kamoun et al., 1998; Fawke et al., 2015; Derevnina et al., 2016). The INF1 elicitin shares 79% amino acid sequence identity with cryptogein. INF1 is shown to bind phytosterols but its essentiality as a sterol carrier for pathogen is not confirmed because the INF1 deficient *P. infestans* can still survive (Kamoun et al., 1998; Fawke et al., 2015). This finding partially conflicts with the traditional definition, where PAMP is defined as an essential contributor to pathogen's fitness (Jones and Dangl, 2006). So far, INF1 is among the most widely used elicitin for understanding molecular mechanisms for the induction of PTI, and for the suppression of PTI by effectors (Kanzaki et al., 2003; Bos et al., 2006; Kawamura et al., 2009; Gilroy et al., 2011b; Cheng et al., 2012; King et al., 2014; Liu et al., 2015; Turnbull et al., 2017; He et al., 2018; Murphy et al., 2018).

OPEL is another unique oomycete-specific PAMP, which was identified in *P. parasitica* and has homologs in several *Phytophthora* spp. and other oomycetes such as *Hyaloperonospora arabidopsidis, Pythium ultimum*, and *Albugo laibachii* (Chang et al., 2015). So far, there is no evidence of its presence in fungi or any other phytopathogens. OPEL is reported to induce strong PTI in tobacco, making it resistant to subsequent infection by *P. parasitica* as well as other diverse pathogens like *Tobacco mosaic virus* (TMV) and *Ralstonia solanacearum* (Chang et al., 2015). OPEL is a 556 amino acid large modular protein consisting of a signal peptide and three conserved domains: a glycine-rich protein domain, a thaumatin-like domain, and a glycosyl hydrolase domain harboring a laminarinase active site. This laminarinase active site is associated with the elicitor activity of OPEL, which is recognized by a PRR either directly or indirectly through DAMPs generated through its enzymatic activity (Chang et al., 2015).

Pep-13 is another PAMP that is apparently unique to *Phytophthora* spp. (Nürnberger et al., 1994). Pep-13 is a highly conserved 13 amino acid peptide found in the cell-wall associated transglutaminases (TGases) of many *Phytophthora* spp. TGases are R-glutaminyl-peptide:amine-γ-glutamyl transferases involved in specific protein cross linking, which are associated with multiple physiological activities in animals (Brunner et al., 2002). No known biological function of TGases in *Phytophthora* spp. has been reported yet other than the induction of PTI in the plants (Reiss et al., 2011; Severino et al., 2014). The *P. sojae*'s Pep-13 was found to induce PTI, strong enough to make parsley a non-host for the pathogen.

### Non-proteinaceous PAMPs of *Phytophthora* spp.

In addition to proteinaceous PAMPs, components of the pathogen's cell wall or membranes that are generated by host enzymes also display PAMP activity. For example, β-glucans are cell wall polymers, which are released from a pathogen's cell wall by host glucanases (Umemoto et al., 1997). β-glucans are common PAMPs of filamentous pathogens (Fesel and Zuccaro, 2016). Different β-glucan oligosaccharides possess conserved characteristics patterns that are recognized by specific hosts. For example, β-1,6-glucans from *P. sojae* can be perceived as PAMPs by soybean but not by tobacco. Phytoalexin biosynthesis in rice and soybean is initiated by different β-1,3- and β-1,6-glucans. Also, different kinds of defense responses are induced by different β-glucans. These characteristics make β-glucans excellent candidates to dissect non-host resistance (Fesel and Zuccaro, 2016; Mélida et al., 2018).

Eicosapolyenoic acids (EPs) are polyunsaturated fatty acids including eicosapentaenoic acid (EPAs), and arachidonic acid (AA). EPs isolated from *P. infestans* were reported to function as PAMPs in a wide variety of plants. Application of low concentration of AAs (isolated from *Phytophthora* and fungi) on potato, tomato, sugar beet, and vine plants enhanced their resistance against a variety of diseases caused by filamentous pathogens (Dedyukhina et al., 2014). A 20-carbon chain and cis-unsaturation at their 5-position in EPs has been reported as the minimum structural features required for their elicitor activity (Preisig and Kuć, 1985). Among plant pathogens only oomycetes, primitive fungi and nematodes have been reported to synthesize these fatty acids. In mammals, EPAs and AAs have been reported to play an important role in mediating inflammatory responses, function of the central nervous system and immune signaling. Their association with both plant and animal immune systems and their presence in multiple pathogens make them putative evolutionary conserved cross-kingdom immune signaling molecules that perhaps evolved by convergent evolution. Another interesting fact about EPs is their potential interaction with β-1,3-glucans, which alone do not induce any defense in potatoes but remarkably enhance the sensitivity of potato tissues to EPs (Bostock et al., 2011).

### *Phytophthora* spp. Elicitors With Conflicted PAMP or Effector Status

In addition to the well-defined oomycete PAMPs, there are some elicitors that qualify to be designated PAMPs as well as effectors. Cell wall degrading enzymes (CWDEs) are one such example. CWDEs have been reported as common elicitors of filamentous pathogens and they contribute to pathogen's virulence.

Plant cell wall is not only an important component of preformed defense that serves as a barrier to prevent pathogen penetration but also as an essential element of plant's surveillance system. PRRs monitor the apoplastic environment and perceive any intrusion in the cell wall integrity by sensing peptides or wall glucans, derived as a result of cell wall damage caused by the pathogen's CWDEs (Bacete et al., 2018). Cell wall damage facilitates pathogen's penetration and DAMPs also serve as nutrients for the pathogen (Ma et al., 2015; Gui et al., 2017). An endoglucanase type CWDEs, XEG1, which is a member of glycoside hydrolase family 12 (GH12), is identified in *Phytophthora* spp. It is defined as PAMP because the GH12 family is widely distributed among prokaryotic and eukaryotic microbial taxa and it is recognized by the host through the PTI recognition machinery. It is also described as an effector because it is an important virulence factor that is secreted in the host's apoplast to damage the host cell wall by hydrolyzing xyloglucan and β-1,4-glucan to reducing sugars (Ma et al., 2015, 2017). The role of CWDEs in the induction of plant defense response has long been established in case of oomycetes but the PAMP status of a fungal CWDE was recently established. The GH12 proteins of *Verticillium dahlia* fungus act as PAMPs as it can trigger PTI in *Nicotiana benthamiana*, independently of their enzymatic activity (Gui et al., 2017).

Carbohydrate-binding module family 1 (CBM1) domain-containing proteins are another type of CWDEs, common among fungi and oomycetes. Although structurally similar, CBM1 proteins of oomycetes and fungi have been reported to play different biological roles during their interaction with the plant cell walls. Cellulose binding elicitor lectin (CBEL), a CBM1-containing protein, first identified in the cell wall of *P. parasitica* is now a well-defined PAMP of *Phytophthora* spp. (Larroque et al., 2013). It can induce PTI in a limited number of species across diverse plant families (Khatib et al., 2004). In addition to triggering PTI, CBEL was found to be involved in maintaining cell wall integrity and pathogen adhesion to the host cell surface but devoid of any enzymatic activity (Séjalon-Delmas et al., 1997). Whereas, in fungi CBM1 containing proteins are enzymatically active and have been shown to be involved in cellulose degradation (Larroque et al., 2012). Interestingly, a fungal CBM1 was recently shown to suppress PTI induced by the GH12 fungal PAMP (Gui et al., 2017), indicating their effector activity in fungi.

Necrosis and ethylene-inducing peptide 1-like proteins (NLPs) are another example of PAMP effector overlap and have been found as common elicitors among the microbes of prokaryotic (bacteria) and eukaryotic (fungi and oomycetes) lineages (Gijzen and Nürnberger, 2006). NLPs were discovered as cytotoxic proteins secreted by necrotrophic and hemi-biotrophic pathogens to initiate necrosis in their dicotyledonous hosts. Later on, non-cytotoxic forms of NLPs were found, secreted by the pathogens during biotrophy (Böhm et al., 2014). NLPs were initially proposed as dual function effector toxins, acting both as virulence factors as well as trigger for ETI (Kamoun, 2006; Böhm et al., 2014). Recently, a highly conserved 20 amino acid fragment referred to as nlp20 found in both cytotoxic and non-cytotoxic NLPs of all three microbial taxa has been shown to incite PTI in various plants. Like XEG1, nlp20 is also recognized through the PTI recognition system. These findings suggest NLPs as unique widespread virulence factors, harboring a highly conserved PAMP motif and have the ability to incite both PTI and ETI (Böhm et al., 2014).

Like CWDEs, NLPs are also mentioned both as PAMPs and effectors in literature without clarification on their specific characteristics behind their respective designation (Haas et al., 2009; Hardham and Cahill, 2010; Böhm et al., 2014; Fawke et al., 2015; Raaymakers and Van Den Ackerveken, 2016). As discussed above, CWDEs and NLPs qualify both as PAMP and effector status and thus, are excellent examples of the PTI-ETI continuum in diverse pathosystems, including *Phytophthora* spp.

### Cognate PRRs and PAMPs Perception Models

**Figure 1:**
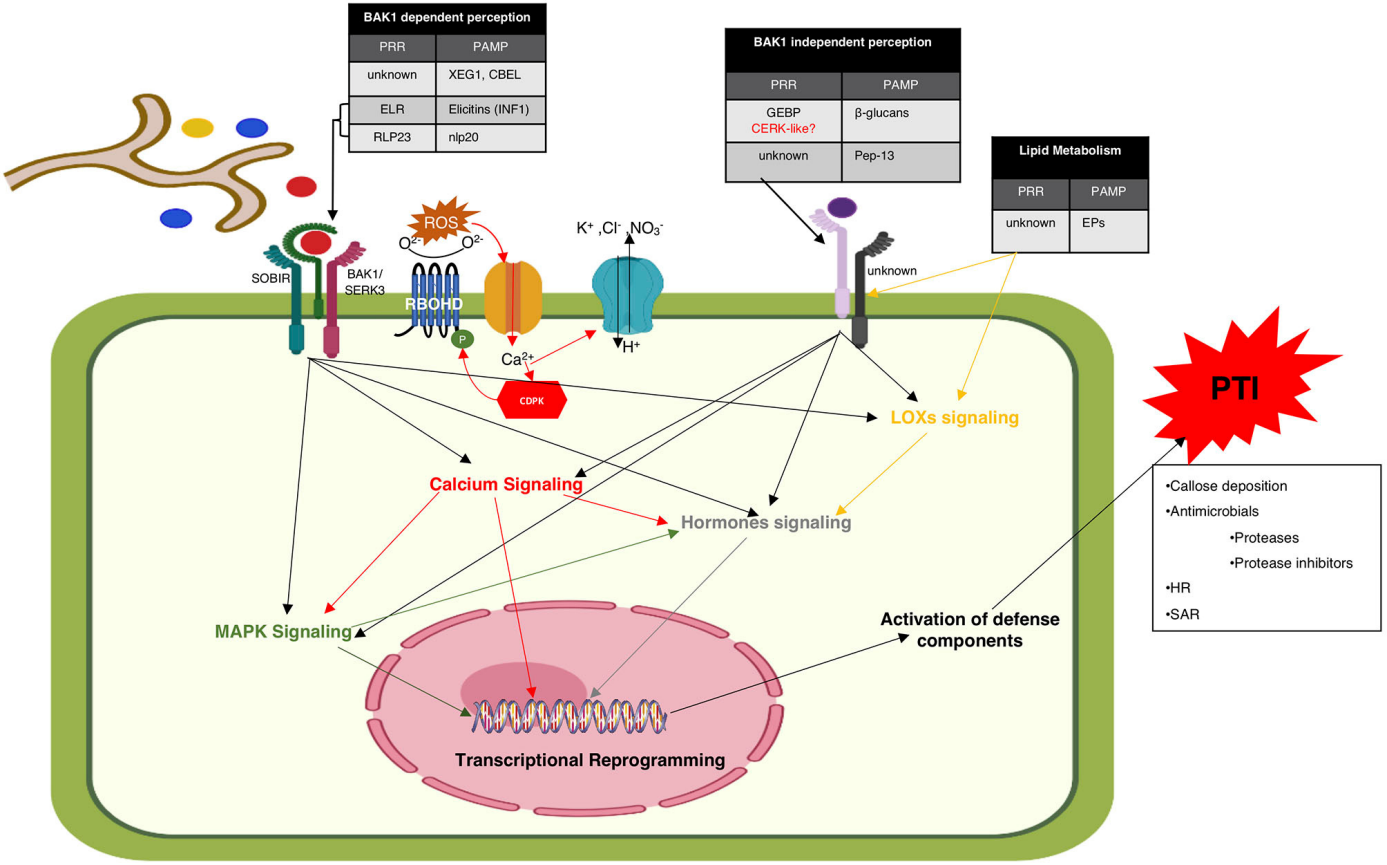
Proposed model depicting the components and events of PAMP triggered immunity. According to the to date literature, there are two PAMPs perception models. One is BAK1/SERK3 dependent (left) and the other one is BAK1/SERK3 independent perception (right). All PAMPs shown in the leftmost table are perceived in BAK1/SERK3 dependent manner. Ca^2+^ ion influx is among the earliest events of PAMP perception followed by opening up the membrane transporters for the influx of H^+^ and efflux of K^+^, Cl^−^, and ${\text{NO}}_{3}^{-}$ that leads to changes in extracellular pH and depolarization of plasma membrane. ROS burst is reported to occur within minutes of PAMP perception. Ca^2+^-dependent or Ca^2+^independent phosphorylated RBOHD generates ROS that leads to further increase in cytosolic Ca^2+^ concentrations. Calcium, MAPK, and hormonal signaling are all reported to be involved in receptor mediated perception of *Phytophthora* spp. PAMPs. PAMPs induce defense responses include; ROS burst, callose deposition, hypersensitive response (HR), expression of defense genes, accumulation of antimicrobial secondary metabolites and systemic acquired resistance (SAR).

Based on bacterial, fungal, and oomycete PAMPs there are two PAMP perception models (Figure 1). The most well-described one is the BAK1/SERK3 dependent perception. BAK1/SERK3 is an LRR repeat receptor-like kinase (LRR-RLK) type cell surface receptor, that was first identified as an interactor of the brassinosteroid receptor 1 (BRI1). Later, it was identified as an essential component of PTI against diverse PAMPs. Induction of HR by INF1 in tobacco and *N. benthamiana* is BAK1/SERK3-mediated (Chaparro-Garcia et al., 2011). Recognition of INF1 by RLP or RLK type PRR and location of INF1-PRR interaction was not clear, but recently it was shown that multiple INFs are perceived by the extracellular domain of a wild potato RLP, elicitin response receptor (ELR), which associates with BAK1 to modulate localized cell death in potato (reviewed in Fawke et al., 2015). The suppressor of BIR1 (SOBIR1) was identified as another required component in ParA1-induced ELR-BAK1 mediated non-host resistance against *P. parasitica* in tomato and *N. benthamiana* (Peng et al., 2015). Wild potato ELR is also reported to recognize cryptogein (Du J. et al., 2015; Derevnina et al., 2016) but association of BAK1 and SOBIR1 with cryptogein induced HR is not clear yet.

The nlp20 PAMP of NLPs has been shown to be recognized by an *Arabidopsis* PRR, RLP23 that interacts with SOBIR1 and BAK1 to trigger various PTI responses (Albert et al., 2015). Both the cell wall associated glycoprotein CBEL and the endoglucanase XEG1 have also been found to require BAK1/SERK3 for PTI induction but their cognate PRRs are not yet known (Larroque et al., 2013; Ma et al., 2015). Evidence of involvement of these PAMP recognition components to induce defense in response to nlp20, XEG1, and CBEL is another solid reason in favor of their dual PAMP/effector designation (Ma et al., 2015).

All the above mentioned proteinaceous PAMPs of *Phytophthora* spp. are perceived in BAK1/SERK3 dependent manner. However, recently it was reported that unlike all known oomycetes PAMPs, perception of Pep-13 is not implicated through BAK1/SERK3 (Wang H. et al., 2019). Pep-13 has been shown to bind to a 100 kD monomeric integral plasma membrane receptor of parsley (Nennstiel et al., 1998; Blume et al., 2000) but its cognate PRR in other plant species is not yet known.

Although known for a long time, the polysaccharide PAMP, β-glucans are far less explored in terms of their host recognition. The PRRs binding β-glucans and the signaling mechanisms involved in β-glucans incited PTI are still unknown. A putative PRR named, GEBP (glucan elicitor binding protein) present on the plasma membrane of soybean was shown to bind *P. sojae*-derived β-1,6-glucan and transduce signals that results in accumulation of phytoalexins (Umemoto et al., 1997). After this finding, many studies were conducted to find β-glucans receptors in other plants. However, these findings are considered controversial because these studies were conducted on β-glucans derived through acidic hydrolysis *in vitro* and currently there is no evidence regarding the binding of plant derived β-glucans to these receptors (Fesel and Zuccaro, 2016). Recently, CERK1, an *Arabidopsis* fungal chitin receptor was reported to bind with synthetic β-1,3-glucan oligosaccharides that are present in both oomycete and fungi. Just like chitin perception via CERK1 (Wang H. et al., 2019), BAK1 was not found involved in this β-1,3-glucan-CERK1 induced PTI responses. Also, the *cerk1* mutants showed higher susceptibility to a biotrophic oomycete *H. arabidopsidis* (Mélida et al., 2018). Thus, providing some bases that CERK1 could be a putative PRR for *Phytophthora* spp. derived β-glucans and their recognition could be independent of BAK1.

EPs are among the earliest discovered elicitors, defined almost 40 years ago. Until now, nothing is known about the cognate PRRs and role of any other member of the PAMP perception model in the recognition of EPs in the host.

### Signaling Mechanisms Involved in Shaping PTI

Upon initial perception of a PAMP by PRR, stereotypic signaling events are initiated, leading to the activation of an array of defense components that collectively formulate PTI (Figure 1). Generally characterized PAMPs induced defense responses include reactive oxygen species (ROS) burst, callose deposition, expression of defense genes and accumulation of antimicrobial secondary metabolites (Larroque et al., 2013). Most of this information is based on the poster child pathosystem, *P. syringae*-*Arabidopsis* interaction. Signaling pathways involved in PTI defense responses induced by *Phytophthora* spp. pathogens are highly fragmented. In the following paragraphs, we have compiled the available information, consistencies and deviations from the general signaling mechanisms considered to be involved in PTI.

#### Calcium Signaling

Calcium signaling is considered as an integral part of receptor mediated perception of most PAMPs of diverse pathogens. Recent studies provide evidence of a complex cross talk between calcium and MAPK signaling pathways. Ca^2+^ ion influx is one of the earliest events that has been reported to trigger within 30 s of PAMP perception. This Ca^2+^ influx opens up the membrane transporters for the influx of H^+^ and efflux of K^+^, Cl^−^, and ${\text{NO}}_{3}^{-}$ that leads to changes in extracellular pH and depolarization of plasma membrane within 1 to 3 min of PAMP perception (Bigeard et al., 2015; Westphal et al., 2019).

PAMP induced Ca^2+^ ion homeostasis in the host has been tested for only a few *Phytophthora* spp. PAMPs mainly because of the experimental limitations. Pep-13 application on transgenic parsley lines expressing aequorin (a Ca^2+^ sensor) represented a biphasic response (Blume et al., 2000). Cytosolic Ca^2+^ concentration was observed to increase after 30 to 40 s of Pep-13 treatment that rapidly peaked at 2 min, and subsequently started decreasing slowly during the next 10 to 40 min to a stable concentration that was almost three times more than the basal levels. The extent of induction in Ca^2+^ influx was found positively correlated with the Pep-13 concentration. Decreased Pep-13 concentration preferentially reduced the transient Ca^2+^ peak, but the sustained increases of cytosolic Ca^2+^ was elicited even at very low Pep-13 concentrations (Blume et al., 2000). Pep-13 has also been reported to induce K^+^ and Cl^−^ efflux, extracellular alkalinization, activation of MAPK and ultimately generation of antimicrobial phytoalexins (Nennstiel et al., 1998; Blume et al., 2000). Elicitins have been reported to trigger calcium and protein kinase mediated signaling leading to the depolarization of plasma membrane, cell wall modifications, protein phosphorylation, potassium (K^+^) and chloride (Cl^−^) efflux and phenylpropanoid metabolism (Bourque et al., 1998; Lochman et al., 2005; Amelot et al., 2011). Induction in nitrate (${\text{NO}}_{3}^{-}$) efflux through anion channels regulated by Ca^2+^ influx and phosphorylation events upstream to cryptogein induced oxidative burst is also reported during cryptogein-tobacco interactions (Wendehenne et al., 2002).

#### Reactive Oxygen Species (ROS)

Generation of reactive oxygen species (ROS) is incited by all discovered *Phytophthora* spp. PAMPs. Extracellular ROS burst is reported to occur within 2 to 3 min after PAMP perception. The respiratory burst oxidase homolog D (RBOHD), a plasma membrane localized NADPH oxidase, mediates ROS induction upon PAMP perception (Bigeard et al., 2015). Regulation of RBOHD during PTI responses has been reported to occur in both Ca^2+^-dependent and Ca^2+^independent manner (Saijo et al., 2018). RBOHD has been reported as an integral component of plasma membrane immune receptor complex involved in antibacterial immunity. It has been shown to exist in complex with both FLS2 and EFR, the most well-studied PRRs of bacterial PAMPs, flagellin, and elongation factor, respectively. The plasma membrane associated kinase BIK1 is a direct substrate of this PRR complex. Upon perception of PAMPs, BIK1 is activated to phosphorylate RBOHD to initiate ROS production in a Ca^2+^-independent manner. Ca^2+^ can directly regulate RBOHD by binding to its N-terminal EF-hand motifs to stimulate ROS production. ROS has been shown to increase cytosolic Ca^2+^ concentrations as well as Ca^2+^-dependent protein kinases (CDPKs) dependent phosphorylation of RBOHD (Kadota et al., 2014; Bigeard et al., 2015). RBOHD association with known PRRs of oomycete and fungal PAMPs has not been reported yet. However, RBOHD has been shown to be involved in CBEL induced PTI responses (except necrosis) that are regulated in BAK1-dependent manner. Both RBOHD and BAK1 have been shown essential in developing *Arabidopsis* root resistance against *P. parasitica* (Larroque et al., 2013). On the other hand, BAK1-independent PTI responses induced upon fungal β-1,3-glucan perception by CERK1 did not require RBOHD to induce PTI responses including ROS burst (Mélida et al., 2018), and the same could be speculated for oomycetes β-glucans. In *N. benthamiana* RBOHA and RBOHB were reported to participate in resistance against *P. infestans* (Yoshioka et al., 2003). Silencing of these genes resulted in enhanced susceptibility of *N. benthamiana* to non-virulent *P. infestans*.

#### PAMP-Induced Hypersensitive Response (HR)

Activation of pathogenesis-related (PR) proteins and other defense components leading to the induction of HR has also been reported. PR proteins respond to cryptogein either in calcium dependent or independent manner (Lebrun-Garcia et al., 1998; Lochman et al., 2005; Dahan et al., 2009). Hoeberichts et al. investigated the effect of light during cryptogein-tobacco interaction and reported that cryptogein induced transcriptional changes are immensely affected by photoperiod and that the HR response is significantly delayed under light (Hoeberichts et al., 2013). Just like cryptogein, INF1 is shown to induce HR through calcium and protein kinase signaling mechanisms. Induction of INF1 induced HR is shown to be independently regulated by calcium and protein kinase signaling pathways. Oxidative burst is incited but not necessarily required for HR. However, it is reported to be involved in both calcium and protein kinase signaling pathways (Sasabe et al., 2000). MAPK interacting cytosolic proteins HSP70 and HSP90 are reported to be the essential components of INF1 induced HR (Kanzaki et al., 2003).

Plant lipoxygenases (LOXs) have also been reported to regulate PAMP induced HR particularly during incompatible host-pathogen interactions. LOXs are the enzymes that catalyze the oxygenation of polyunsaturated fatty acids to produce hydroperoxides that serve as precursors of different biologically active compounds with distinct physiological functions as well as in plant defense responses. Plant LOX precursors can be divided into two major classes; 9-LOXs and 13-LOXs according to the position on the carbon chain at which oxygen is incorporated into linoleic acid or linolenic acid, the lipid substrates for LOX catalysis in plants (Ricker and Bostock, 1994; Kolomiets et al., 2001). Distinct LOX mediated products have been suggested to be involved in plant-pathogen interactions in different ways; 9-LOXs are speculated to cause oxidative damage to plant membranes during HR and serve as direct antimicrobial agents whereas, 13-LOXs are reported to act as signaling molecules in Jasmonic acid (JA) metabolism (Slusarenko et al., 1993; Hwang and Hwang, 2010).

Discovery of lipid PAMPs, EPs as *Phytophthora* spp. elicitors first led to the hypothesis about the role of lipid metabolism in early stages of plant-pathogens interactions (Véronési et al., 1996). Since then, LOX activity has been widely monitored during plant-pathogen interactions and is reported to be positively correlated with plant resistance against pathogens of different lineages (Véronési et al., 1996; Kolomiets et al., 2001; Hwang and Hwang, 2010; Afroz et al., 2013). *Phytophthora* spp. pathogens and even their non-lipid PAMPs have been reported to enhance LOX activity in the host cells (Véronési et al., 1996). The Ca^2+^dependent TGase PAMP, Pep-13 showed enhanced LOX expression in potato cells. CBEL treated tobacco cell suspension and the whole plant, both showed up to 2.5-folds increase in LOX activity (Séjalon et al., 1995). Although known for a long time the pathway behind LOX related plant defense responses is not defined, except some fragmented clues generated from different pathosystem studies. The 9-LOX hydroperoxides generation upon cryptogein treatment has been reported as a crucial element for HR in tobacco (Rustérucci et al., 1999).

#### PAMP-Induced Systemic Acquired Resistance (SAR)

In addition to localized resistance, various *Phytophthora* spp. PAMPs are shown to trigger the transport of defense signals throughout the plant that results in broad-spectrum disease resistance against secondary infections. This spread of resistance across the plant is known as systemic acquired resistance (SAR) (Gao et al., 2015). For example, EPs can induce SAR in different plant species, which is effective against subsequent infection by diverse pathogens (Savchenko et al., 2010; Robinson and Bostock, 2015). In tobacco, treatment of lower leaves with AA was shown to induce SAR against TMV in the upper leaves of the plants. Transgenic *Arabidopsis* expressing EPs showed broad spectrum resistance against diverse pathogens including *P. capsici* (oomycete), *B. cinerea* (fungi), and aphids (insect), but were highly susceptible to *Pseudomonas syringae* pv *tomato* (bacteria). Increased transcription of JA related genes as well as enhanced JA levels were observed in these EP plants, whereas salicylic acid (SA) mediated signaling was found compromised (Savchenko et al., 2010). These findings are consistent with the general assumption that SA and JA signaling pathways are antagonistic to each other. In contrast, both SA and JA pathways were found to be involved in the induction of Pep-13 mediated SAR in potato (Halim et al., 2009). In tomato, INF1 infiltrated in the middle of fully expanded leaves was found to activate SAR against *Ralstonia solanacearum* in the distal parts of the challenged leaves (Kawamura et al., 2009). Jasmonic acid (JA) and ethylene (ET) signaling (but not SA) pathways were found in this SAR development. Conversely, cryptogein induced SAR in tobacco was SA mediated, which was found effective against *P. parasitica* var *nicotianae* (Wendehenne et al., 2002). OPEL-induced SAR in systemic leaves was found effective against cross kingdom pathogens like, TMV (virus), *Ralstonia solanacearum* (bacteria), and *P. parasitica*. Enhanced expression of several SA-responsive genes and lipid transfer protein DIR1 was observed in tobacco plants in response to OPEL treatment that suggests SA and/or DIR1-mediated SAR. Further investigation is required to confirm the involvement of these signaling molecules in OPEL-induced SAR (Chang et al., 2015). XEG1 induced SAR in soybean and *N. benthamiana* was found effective against *P. sojae* and *P. parasitica*, respectively (Ma et al., 2015). The signaling mechanisms behind the development of this SAR are not investigated yet. NLPs are generally characterized as ethylene (ET) inducing proteins (Böhm et al., 2014). Treatment of *Arabidopsis* with nlp20 protected the plant from subsequent infection with virulent isolates of *Pseudomonas syringae* pv. *tomato, Botrytis cinerea*, or *H. arabidopsidis* (Albert et al., 2015). Enhanced expression of SA related genes has been found in *Arabidopsis* upon treatment with HaNLP3 that is nlp20 homolog in a non-*Phytophthora* oomycete *H. arabidopsidis* (Oome et al., 2014). Specific signaling mechanisms behind nlp20 induced SAR needs further investigations. CBEL induced SAR was not specifically tested but unlike other PAMPs that activate selective phytohormones, CBEL has been shown to induce all three hormone signaling pathways; SA, JA, and ET that are differentially involved in the induction of necrosis and other defense responses in *Arabidopsis* (Khatib et al., 2004). Despite being one of the earliest discovered elicitors, signaling mechanisms behind β-glucan induced PTI are still not known. Crude β-glucan (Kopp et al., 1989) preparations from *P. sojae* and *P. infestans*, elicited systemic protection against different viruses in soybean and various *Nicotianae* spp. (Kopp et al., 1989). Application of different β-glucan preparations from *P. sojae* and brown alga *Laminaria digitate* showed accumulation of PR genes and SA in tobacco leaves. SAR was only tested for laminarin from *L. digitate*, which was found effective against soft rot bacterium *Erwinia carotovora* (Klarzynski et al., 2000).

Conclusively, upon recognition by cognate plant PRR, *Phytophthora* spp. PAMPs/elicitors can trigger PTI through crosstalk among several interlinked and independent defense signaling components. Point to be noted is, HR and SAR are generally considered as ETI responses but as discussed above *Phytophthora* PAMPs are reported to induce both localized (HR) and systemic (SAR) resistance that can protect the plant against diverse range of pathogens.

## Effector Triggered Susceptibility (ETS)

**Figure 2:**
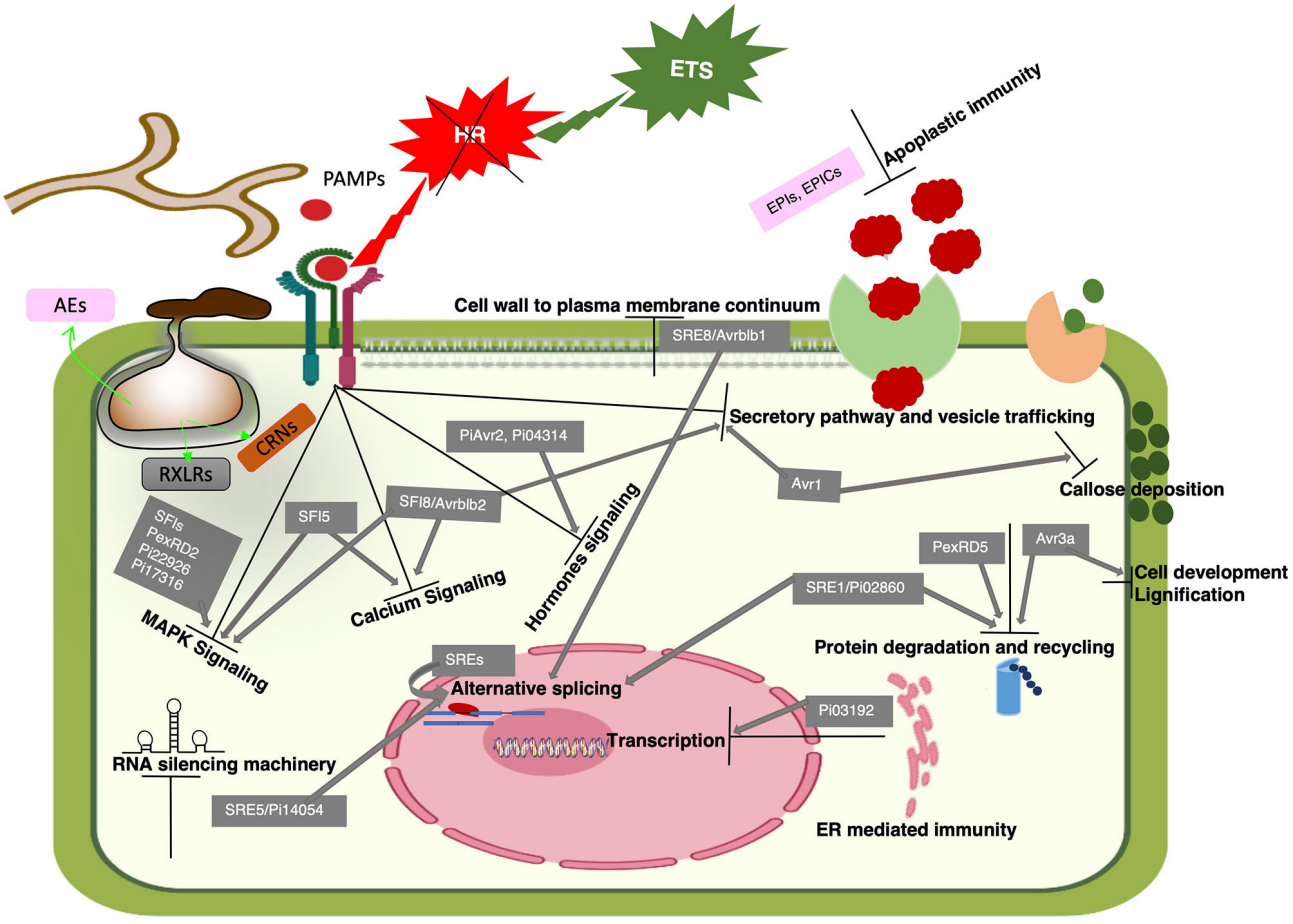
A proposed model of PTI to ETS transition. *Phytophthora* spp. deploy multiple effectors to target various components of the host's signaling pathways, metabolic and physiological machinery to promote infection. Here we have only shown *P. infestans* effectors known to modulate defense responses in favor of infection. An immune component is intervened at different levels by multiple effectors acting at different locations. For example, apoplastic immunity is modulated by the action of pathogen's protease inhibitors (EPIs, EPICs) against host's proteases in the apoplast and RXLR (Avr1, Avrblb2) effectors operate intracellularly to prevent the secretion of host's proteases into the apoplast. Some effectors target multiple host proteins to interfere with different signaling pathways and also one signaling component is targeted by several effectors. For example, SFI8 interferes with calcium signaling, inhibits protease secretion into the apoplast and interferes with MAPK signaling. MAPK signaling is targeted by 11 RXLR effectors including eight SFIs. Alternative splicing is modulated by nine SREs and, SRE 1, 5, and 8 are known to target proteolysis, RNAi and cell wall to plasma membrane continuum, respectively. Most of these effectors (details in Table 2) have shown to overcome INF1 induced HR thus, indicate PTI suppression in various ways to establish ETS.

In order to establish infection, successful *Phytophthora* pathogens have devised ways to either escape recognition or circumvent host immunity (Figure 2). In accordance with the zigzag model, *Phytophthora* spp. carry myriads of effector proteins that are secreted inside the host cells to modulate the PTI and to trigger ETS (Jones and Dangl, 2006). *P. parasitica* carries INF1 like elicitin called ParA1 that is recognized by tobacco to induce defense (Kamoun et al., 1993). It has been found that virulent strains of *P. parasitica* can bypass this recognition by suppressing ParA1 expression during their interaction with compatible hosts, and this adaptation enables it to cause black shank disease in tobacco (Colas et al., 2001). Since sequence divergence was not observed in *ParA1* gene, this expression modulation could be the result of the effector activities during infection. The role of *P. parasitica* effectors in *ParA1* downregulation is not investigated, but several RXLR effectors from different *Phytophthora* spp. had been reported to suppress INF1-induced cell death (Bos et al., 2006; Gilroy et al., 2011b; Wang et al., 2011; He et al., 2018; Murphy et al., 2018).

**Figure 3:**
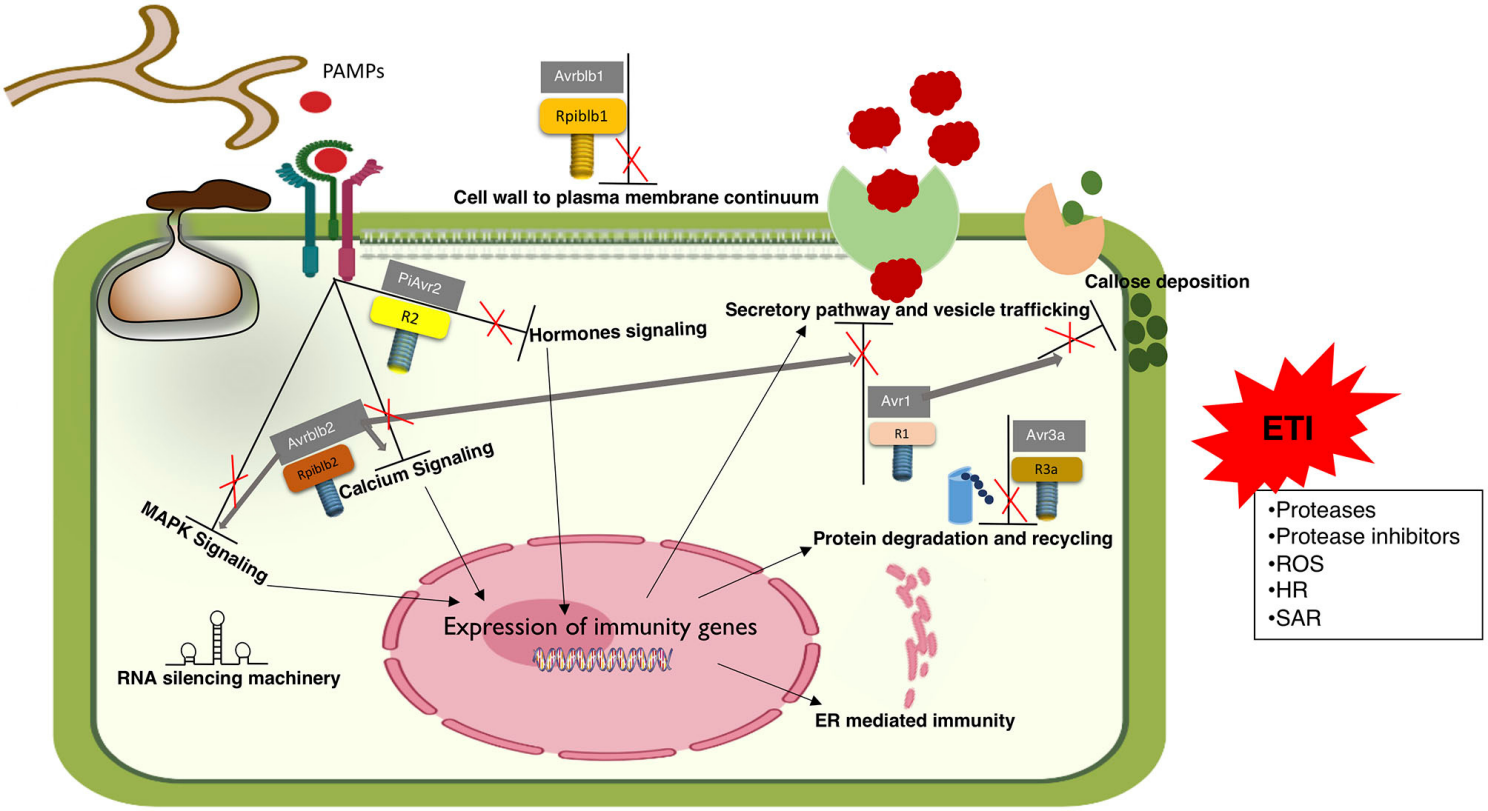
*R genes* mediated transition of ETS to ETI. Proposed model depicting the activation of ETI through recognition of effectors by cognate *R genes*. Although numerous effectors are known to establish ETS by modulating all known defense responses, little is known in terms of ETI. Very few *R genes* are known to identify their cognate effectors. Mechanisms behind this recognition are also scarcely known. In agreement to the zigzag model, ETI responses are just prolonged and robust PTI responses.

*Phytophthora* spp. effectors can be broadly categorized into two classes with respect to their localization: apoplastic effectors that are secreted in the apoplast of the host cells and cytoplasmic effectors that are translocated inside the host cells. Apoplastic effectors are diverse hydrolytic enzymes like glycoside hydrolases (GHs), pectinases, proteases, and protease inhibitors (Mcgowan and Fitzpatrick, 2017). Based on conserved motifs, cytoplasmic effectors of *Phytophthora* spp. are categorized in two groups, the RXLR effectors with RxLR dEER (Arg, any amino acid, Leu, Arg) motif and crinklers (CRNs) having LxFLAK motifs. *Phytophthora* spp. carry 300-700 RXLR effectors with almost no sequence similarity to known proteins (Guo et al., 2019). The characteristic N-terminal RXLR motif is implicated in the translocation of effectors inside host cells.The C-terminal portion of RxLR effectors, which has different amino acid sequences, carry the PTI-suppressing and disease promoting effector activity in susceptible plants and the R-activating avirulence activity in resistant plants (Figures 2, 3 and Table 2) (Birch et al., 2008; Bozkurt et al., 2012; Anderson et al., 2015).

**Table 2:** *Phytophthora* spp. effectors that modulate host defense responses to establish ETS or trigger ETI.

***Phytophthora* spp**.	**Host species**	**Effector**	**Host target**	**Specific elicitor induced cell death suppression**	**Virulence function to establish ETS**	**ETI and *R* gene**	**References**
*P. infestans*	*N. benthamiana*	Avr1	Sec5 Exocyst subunit	CRN2	Prevent PR-1 secretion and callose deposition	YesR1	Du Y. et al., 2015; Du et al., 2018
		Avrblb2/SFI8	C14 Protease		Prevent C14 secretion	YesRpiblb2	Bozkurt et al., 2011
	Tomato CaMs tested in *N. benthamiana*		CaMs		Interferes with calcium signaling to regulate ETI		Naveed et al., 2019
	Tomato *Arabidopsis*		Unknown		Interferes with MAPK signaling		Zheng et al., 2014
		SFI1, SFI2, SFI5, SFI6, SFI7					
	Potato	SFI3	UBK				He et al., 2019
		SFI5	CaMs		Interferes with calcium signaling to suppress PTI		Zheng et al., 2018
	Potato *N. benthamiana*	PexRD2	MAP3Kε	INF1	Interferes with MAPK signalig		King et al., 2014
		Pi22926	MAP3Kβ2	Avr4/Cf4 and AvrPto/Pto associated HR			Ren et al., 2019
	Arabidopsis *N. benthamiana*	Avrblb1/IPI-O1/SRE8	LecRK-1.9 Lectin receptor kinase		Disrupt cell wall to plasma membrane continuum	YesRpi-blb1	Bouwmeester et al., 2011
	Potato *N. benthamiana*	Pi03192	NAC transcription factors		Prevent NAC localization from ER to nucleus	No	Mclellan et al., 2013
	Tobacco *N. benthamiana*	Avr3a	CMPG1 E3 ligase	INF1	Prevent ubiquitin-dependent proteolysis	YesR3a	Bos et al., 2006; Gilroy et al., 2011b
		PexRD54	ATG8CL autophagy protein		Modulate autophagy mediated defense		Dagdas et al., 2016, 2018
	*N. benthamiana*	SRE3	U1-70K		Modulate Alternative splicing		Huang et al., 2020
		Pi14054/SRE5	Unknown		Suppressor of RNA silencing		Vetukuri et al., 2017
	Potato	PITG_15718.2	Unknown		Attenuate plant defense and growth		Wang J. et al., 2019
**S-factors**							
	*Arabidopsis N. benthamiana*	PiAvr3a	CAD7		Suppress lignification		Li T. et al., 2019
	Potato *N. benthamiana*	Pi02860	NRL1 E3 ligase	INF1	Enhance proteasome-mediated degradation of SWAP70		He et al., 2018
	*N. benthamiana*	Pi04089/SRE2	KRBP1 RNA-binding protein		Possibly affects post translational modifications		Wang et al., 2015
	Potato *N. benthamiana*	Pi17316	StVIK (MAP3K)	INF1	Interferes with MAPK signaling		Murphy et al., 2018
	Potato *N. benthamiana*	PiAvr2	BSL family	INF1	Interferes with brassinosteroid (BR) hormone signaling	YesR2	Gilroy et al., 2011a; Turnbull et al., 2017, 2019
	Potato *N. benthamiana*	Pi04314	PP1cs		Modulate JA and SA signaling		Boevink et al., 2016b
*P. sojae*	soybean	Avh240	AP1 Protease		Prevent AP1 secretion		Guo et al., 2019
		PsAvh238	ACSs		Suppress ET biosynthesis		Yang et al., 2019
		PSR1 and PSR2	PINP1 RNA helicase		Suppress RNA silencing		Qiao et al., 2013, 2015
	*Arabidopsis* and *N. benthamiana*	Avh331		INF1	Interferes with MAPK signaling		Cheng et al., 2012
	*Arabidopsis*	PsCRN63 (crinkler)		flg22			Li et al., 2016
	*N. benthamiana*	PsAvh262	BiPs (S-factors)		Suppress ER mediated immunity		Jing et al., 2016
*P. capsici*	*Arabidopsis*	Avr3a12	FKBP15-2		Inhibits ER stress sensing		Fan et al., 2018
		PcAvh103	EDS1		Suppress SA mediated defense		Li et al., 2020
		RxLR48	NPR1				Li T. et al., 2019
		PSR2	RNA binding protein DRB4		Modulates RNA silencing		Hou et al., 2019
		RxLR207	ACD11, BPA1 and BPLs (S-factors)		Supress ROS-mediated defense		Li T. et al., 2019
	*N. benthamiana*	Avh1	PP2Aa scaffolding subunit		Attenuate plant defense and growth		Chen et al., 2019

The blurriness of effector vs. PAMP terms is found more in case of apoplastic effectors (described in further sections). Cytoplasmic effectors on the other hand show less conflict of being designated effectors. In general, in contrast to cytoplasmic effectors, apoplastic effectors tend to behave either like PAMPs or effectors. In literature, some authors referred to CRNs as PAMPs, whereas others as effectors (Torto et al., 2003). Torto et al. discussed the possibility of CRNs as *Phytophthora* spp. PAMPs based on some observations pointing toward the similarities of CRNs with the then well-known PAMP, INF1 but the authors have clearly stated, “whether the CRN proteins function as PAMPs remains to be determined” (Torto et al., 2003). Based upon the existing evidence, CRNs fit well in the effector category (Wang et al., 2018; Maximo et al., 2019).

Identification of the host's targets hijacked by the pathogen's effectors and characterizing their role in the infection development and defense manipulation has immensely contributed to our understanding of the molecular basis of plant-pathogen interactions. Based on current research, it has been found that effectors from diverse pathogens target conserved cellular processes across diverse plant families. Following the norm, *Phytophthora* spp. effectors have also been found to target cellular processes targeted by bacterial and fungal pathogens across different plant hosts (Toruño et al., 2016). Immune suppression by *Phytophthora* pathogens can be achieved in two ways: (1) by inhibiting the activity of positive regulators of immunity or (2) by supporting the function of negative immune regulators. Till now, all the apoplastic effectors have been found targeting the positive immune regulators, whereas cytoplasmic effectors particularly, the RXLR effectors have been found targeting both the positive and negative regulators of defense involved in different cellular processes at various subcellular locations.

### *Phytophthora* spp. Effectors Targeting Positive Immune Regulators

#### Modulation of Apoplastic Immunity by the Apoplastic Effectors

Plant's apoplast serves as a battleground for plant-pathogen warfare. Either as preformed defense or upon PTI activation, plants secret several diverse catalytic proteins to the apoplast against attackers. Pathogens deploy a wide variety of apoplastic effectors that compromise host defense proteins as well as cellular components to facilitate pathogen colonization and penetration into the host cells. As described above, many of these *Phytophthora* spp. apoplastic effectors are considered as PAMPs because they are conserved across the genus. For example, PsXEG1, a glycoside hydrolase of *P. sojae*, is secreted in the host's apoplast to damage the cell wall by reacting with specific glucans. A very interesting defense and counter defense strategy in the soybean-*P.sojae* system was discovered recently. A soybean glucanase inhibitor protein, the GmGIP1, has been shown to bind PsXEG1 in the apoplast and block its virulence activity. To counter this defense, *P. sojae* deploys a paralog of PsXEG1 named as PsXLP1(PsXEG1-like protein). PsXLP1 lacks endoglucanase activity but it can bind to GmGIP1 with far more affinity than PsXEG1, thus protecting PsXEG1 from inhibitory effect of GmGIP1 and freeing it for its virulence activity. Similar role of XEG1-XLP1 has been found to contribute to *P. parasitica* virulence in *N. benthamiana*. Many other *Phytophthora* spp. have been reported to carry this gene pair suggesting that XLP1 deployment to guard XEG1 against apoplastic glucanase inhibitors could be a conserved counter defense strategy across the genera (Ma et al., 2017). On the contrary to the above example where soybean produces glucanase inhibitor (GmGIP1) specifically against *P. sojae's* glucanase (XEG1), *P. sojae was* also found to secrete a specific glucanase inhibitor effector, PsGIP1, to inhibit host's endo-β-1,3-glucanases to prevent the degradation of β-1,3/1,6-glucans in the pathogen's cell wall and thus avert the release of defense-eliciting oligosaccharides (Damasceno et al., 2008).

*Phytophthora* spp. have also been reported to carry specific extracellular protease inhibitors (EPIs) against host's protective proteases in the apoplast. Different *Phytophthora* spp. protease inhibitors have also been found targeting different classes of secreted host proteases primarily subtilisin-like serine proteases (Figueiredo et al., 2018) and papain-like cysteine proteases (PLCPs) (Misas-Villamil et al., 2016). *P. infestans* secrets Kazal family serine protease inhibitors, EPI1 and EPI10, into the host's apoplasts where they interact with P69B, a tomato subtilisin-like serine protease (PR-7 family) secreted to the host's apoplast (Tian et al., 2004, 2005). Another class of *P. infestans* effectors is cystatin-like protease inhibitors, EPIC1 to EPIC4. Among these, EPIC1 and EPIC12B can interact and interfere with different host papain-like cysteine proteases (PLCPs). Tomato plants carry seven PLCPs, three out of these seven; C14, ${\text{Rcr}}_{,}^{3\text{pim}}$ and *Phytophthora*-inhibited protease 1 (Pip1) that is also classified as PR protein closely related to Rcr^3pim^ (Kaschani et al., 2010) have been found to interact and inhibited by both EPIC1 and EPIC12B in the apoplast of the host. These three PLCPs, C14, Rcr^3pim^, and Pip1 (3-PLCPs) are also targeted by fungal and bacterial effectors (Kaschani et al., 2010; Shindo et al., 2016), and thus can be speculated as hubs of apoplastic immunity targeted by pathogens of diverse lineages.

#### Modulation of Vesicle Trafficking and Secretion

In addition to modulation of apoplastic immunity by the apoplastic protease inhibitor effectors in the apoplast, some RXLR effectors are reported to interfere with plant secretory pathway and vesicle-trafficking intracellularly, to suppress the secretion of proteases and other antimicrobial compounds to the apoplast. For example, a *P. infestans* RXLR effector, Avr1 is reported to interact with Sec5, a subunit of exocyst complex to hijack exocytosis. Avr1 was found to be localized with Sec5-associated mobile bodies around perihaustorial membrane in the host cell (Wang et al., 2018), most probably to stop it from becoming a part of the exocyst complex. Sec5 has been found as a required component for PTI, and Avr1-Sec5 interaction is suggested to interfere with PR-1 secretion as well as callose deposition, thus attenuating host defense against *P. infestans* (Du Y. et al., 2015). Another highly conserved effector, RxLR24 is reported to interact with the members of RABA GTPase family putatively, involved in vesicular secretion of core antimicrobials like PR-1 and defensin (PDF1.2) to promote infection (Tomczynska et al., 2018). The *P. infestans* RXLR effector, PiAvrblb2 (PITG_04090), interacts with C14 protease at the plasma membrane and blocks its secretion into the apoplast of host leaves (Bozkurt et al., 2011). Similarly, another RXLR effector, PsAvh240, has been shown to interact with an aspartic protease, GmAP1, at the soybean plasma membrane to inhibit its secretion into the apoplast and thereby suppressing the apoplastic defense against *P. sojae* (Guo et al., 2019).

#### Modulation of Cell-to-Cell Trafficking

In higher plants, cells are connected through symplastic cell-to-cell channels called plasmodesmata (PD) that enable intercellular trafficking of nutrients and signaling molecules. This intercellular transport is regulated by adjusting the size exclusion limit (SEL) of PD that mainly depends upon callose depositions around the PD openings (Tomczynska et al., 2020). Callose deposition upon PAMP perception is crucial for host defense against *Phytophthora* pathogens (Chang et al., 2015; Du Y. et al., 2015; van den Berg et al., 2018). PD mediated cell-to-cell movement of *P. ramorum* hyphae was reported previously (Giesbrecht et al., 2011). A *P. brassicae* RXLR effector, RxLR3 is shown to inhibit callose deposition at PD by targeting host's callose synthases (CalS) to promote cell-to-cell conductivity that resulted in enhanced infection (Tomczynska et al., 2020). These findings suggest that *Phytophthora* spp. can modulate PD openings to physically spread across neighboring cells during infection.

#### Modulation of Host's Cell Wall to Plasma Membrane (CW-PM) Continuum

Another strategy used by filamentous pathogens to interfere with plant defense is the disruption of cell wall to plasma membrane (CW-PM) continuum in hosts. Adhesion of plant cell wall and plasma membrane is critical for cell wall sensory signaling and induction of several PTI responses like callose deposition, ROS burst and cell death. Peptides containing RGD (R: arginine; G: glycine; D: aspartic acid) motif are well-known to disrupt CW-PM adhesions in plants (Mellersh and Heath, 2001). Avrblb1 (IPI-O1), a *P. infestans* RXLR effector that also contain an RGD motif has been reported to disrupt CW-PM adhesion by targeting a plasma membrane associated lectin receptor kinase, LecRK-I.9. Loss of function mutants of *LecRK-I.9* showed enhanced susceptibility to different *Phytophthora* pathogens (Bouwmeester et al., 2011).

#### Modulation of Endoplasmic Reticulum (ER)Mediated Immunity

Endoplasmic reticulum (ER) quality control system is the critical component involved in shaping PTI responses. Several RXLR effectors have been found to suppress this ER mediated immunity in multiple ways. For example, a *P. infestans* RXLR effector Pi03192 targets two *N. benthamiana* NAC transcription factors at ER membrane and prevent their localization to the nucleus thus, resulting in increased host susceptibility to *P. infestans* by preventing transcriptional regulation of defense components. These NAC TFs are induced by *P. infestans* but not in response to bacterial flg22 indicating their specificity in PTI induced by PAMP of diverse pathogens (Mclellan et al., 2013). ER stress sensing is a crucial component of ER-mediated plant immunity. Recently, a *P. capsici* RXLR effector PcAvr3a12 (Avr3a class), was found to interact with an ER stress sensing protein FKBP15-2 encoding a peptidyl-prolyl cis-trans isomerase (PPIase). This PcAvr3a12- FKBP15-2 interaction inhibits FKBP15-2 stress sensing activity thus, attenuating ER mediated defense response against *P. capsici* (Fan et al., 2018).

#### Modulation of Host's Alternative pre-mRNA Splicing by *Phytophthora* Pathogens

Alternative splicing (AS) of pre-mRNA to generate multiple transcripts of a gene is an essential post-transcriptional process that regulates numerous physiological processes including plant immunity. Recently, Huang et al. have put forth AS as an independent layer of host gene regulation in response to pathogens, and *P. infestans* has been shown to reprogram the AS of host's pre-mRNAs in favor of infection, putatively, by enhancing the production of host's transcripts that promote disease as well as by antagonizing the generation of core host defense transcripts (Huang et al., 2020). For example, PKFP gene that encodes a protein kinase, undergoes AS to generate PKFP.1, a functional isoform that positively regulates plant immunity and PKFP.2, a non-function isoform. *P. infestans* has been found to suppress the generation of PKFP.1 to enhance host's susceptibility. Like other host's physiological processes, modulation in AS by *P. infestans* is implemented by deploying effectors and nine RXLR effectors are presented as splicing regulatory effectors (SREs 1-9). Three of these SREs (3,6, and 7) were shown to bind with core host splicing factors (SFs). SRE3 was shown to modulate host immunity by physically interacting with U1-70K, a core spliceosomal component (Huang et al., 2020). Some of these SREs have been previously reported to target different host's proteins to modulate other physiological processes. For example, SRE1 (Pi02860) was reported to interact with NRL1 to modulate ubiquitin-mediated proteolysis (He et al., 2018), SRE2 (Pi04089) binds with KRBP1, which is presumed to contribute to AS, SRE5 (Pi14054) is a suppressor of host's RNA silencing (Vetukuri et al., 2017) and SRE8 (Pi21388/IPI-O1/Avrblb1) is reported to disrupt host's CW-PM continuum (Bouwmeester et al., 2011). Since, AS regulates almost all physiological responses, deployment of multiple effectors might be the pathogen's strategy to reprogram the host's protein profiling in pathogen's favor.

#### Modulation of RNA Interference Mediated Immunity

Host's RNA interference (RNAi) is well-known for antiviral defense, but its implications in defense against eukaryotic phytopathogens have just begun to unfold. Viral pathogens carry suppressors of RNA silencing to counter host's RNA silencing for successful infection. In 2013, Qiao et al. reported two *P. sojae* RXLR effectors, termed as *Phytophthora* Suppressor of RNA Silencing 1 and 2 (PSR1 and PSR2) that enhanced plant susceptibility by suppressing RNA silencing machinery (Qiao et al., 2013). PSR1 is reported to target a host RNA helicase PINP1 (PSR1-Interacting Protein 1) that regulates the accumulation of endogenous small interfering RNAs (siRNAs) and microRNAs in *Arabidopsis* (Qiao et al., 2015). Recently, Hou et al. (2019) presented an example of trans-kingdom RNAi during *Phytophthora*-*Arabidopsis* interaction. They have shown the induction of endogenous secondary siRNAs in *Arabidopsis* in response to *P. capsici*. Some of these siRNAs were found to target *P. capsici* transcripts for potential gene silencing to mitigate infection. PSR2 was found to counter this host induced pathogen's gene silencing by associating with a core component of host's RNA silencing machinery, a double stranded RNA binding protein (DRB4) that is known to be involved in the biogenesis of secondary siRNAs (Hou et al., 2019). A *P. infestans* effector, Pi14054, has been shown to act as a suppressor of RNA silencing in *N. benthamiana* (Vetukuri et al., 2017). These findings suggest that *Phytophthora* spp. deploy effectors to modulate cross-kingdom RNAi mechanisms, which play a role in host defense.

#### Modulation of Proteolysis and Autophagy Mediated Defense

Protein degradation and recycling either through proteasome or autophagy has been shown to play key roles in plant immunity (Üstün et al., 2016; Hofius et al., 2017). E3 ligases are known to ubiquitylate protein substrates for ubiquitin-dependent proteolysis by the 26S proteasome (Furniss et al., 2018; Serrano et al., 2018). A *P. infestans* RXLR effector Avr3a suppresses INF1 induced PTI by interacting and stabilizing the U-box E3 ligase CMPG1 that functions in protein degradation and is essential component of INF1 induced cell death (Bos et al., 2006; Gilroy et al., 2011b). Recently, *P. infestans* is reported to influence the alternative splicing of some core components of ubiquitin-dependent proteolysis. SP1 gene encodes an E3 ubiquitin protein ligase that undergoes AS to generate SP1.1, a functional isoform that is a positive regulator of plant immunity, and SP1.2, a non-function isoform. *P. infestans* was found to suppress the generation of SP1.1 to enhance host's susceptibility (Huang et al., 2020).

Autophagy has been shown to play a crucial role in shaping HR (Hofius et al., 2017). A *P. infestans* RXLR effector, PexRD54 binds to host autophagy protein ATG8CL to interfere with the formation of autophagosomes. This PexRD54-ATG8CL binding outcompetes ATG8CL binding with an autophagy cargo receptor Joka2 that plays a positive role in defense (Dagdas et al., 2016). They have shown that during *P. infestans* infection, defense-related autophagosomes labeled by ATG8CL-Joka2 are diverted toward perihaustorial membranes, where PexRD54 also localize with these autophagosomes. Moreover, overexpression of ATG9, the protein involved in early autophagosome biogenesis, enhances plant defense (Dagdas et al., 2018). These findings reveal complex modulations in the host's proteolysis and recycling mechanisms during the *Phytophthora*-plant interactions.

#### Modulation of MAPK and Calcium Signaling Pathways by RXLR Effectors

Recent investigations have shown that *Phytophthora* spp. modulate MAPK and calcium signaling pathways by deploying specific effectors that target core components of these signaling pathways (Figure 2). The MAPK signaling plays important roles in both PTI as well as ETI. The MAPK signaling includes three protein kinases: MAP kinase (MAPK), MAP kinase kinase (MAP2K) and MAP kinase kinase kinase (MAP3K). In a typical MAPK signaling cascade, the MAPKs are phosphorylated by MAP2Ks, which themselves are phosphorylated by MAP3Ks (Saijo et al., 2018). Recently, multiple *P. infestans* RXLR effectors have been shown to modulate immunity by suppressing MAPK signaling either by directly interacting with different MAP3Ks components or by targeting the upstream players of MAPK pathways (Ren et al., 2019).

For example, a *P. infestans* RXLR effector PexRD2 binds with the kinase domain of MAP3Kε in the cytosol to interrupt the associated MAPK signaling (King et al., 2014). Overexpression of MAP3Kε results in enhanced phosphorylation of MAPKs and also induce cell death in the plant tissues. PexRD2 is shown to inhibit both MAP3Kε triggered cell death and MAPKs phosphorylation, whereas it was unable to suppress INF1 mediated HR. The HR associated with the recognition of *Cladosporium fulvum* effector Avr4 mediated by the tomato Cf4 protein and the *P. syringe* effector AvrPto by Pto/Prf proteins are known to be MAP3Kε dependent. PexRD2 suppressed both these Avr4/Cf4 and AvrPto/Pto associated HR. On the contrary, it was unable to suppress the R proteins mediated HR triggered by diverse *P. infestans* effectors, AVR3a (RXLR) and CRN8 (crinkler), providing evidence that HR associated with AVR3a/R3a and CRN8/R protein is independent of MAP3Kε (King et al., 2014). Recently, Ren et al. reported another *P. infestans* RXLR effector, Pi22926 that interacts with a different MAP3K of potato, StMAP3Kβ2 in the nucleoplasm. Just like PexRD2, Pi22926 also inhibited Avr4/Cf4 and AvrPto/Pto associated HR but not the INF1 and AVR3a/R3a induced HR, indicating that Pi22926 and PexRD2 may suppress the same specific MAP3K dependent signaling pathway to promote disease. But further investigations revealed a further specification in MAPK signaling modulated by these two effectors. It was found that cell death responses triggered by NbMAP3Kβ2 and NbMAP3Kε are independent of each other and are specifically suppressed by Pi22926 and PexRD2, respectively. These findings suggest that *P. infestans* deploy PexRD2 and Pi22926 effectors at two different subcellular locations (cytoplasm and nucleus, to target two different MAP3Ks that act in parallel in MAP3K-dependent defense signaling induced upon the recognition of unidentified PAMP/PRR or effector/R protein pairs (King et al., 2014; Ren et al., 2019).

Another group of *P. infestans* RXLR effectors called *s*uppressors of the early *f* lg22-induced *i*mmune response (SFIs) were found to suppress flg22 mediated PTI by targeting multiple steps of MAPK signaling (Zheng et al., 2014). All eight SFIs significantly suppressed activation of a flg22 induced PTI reporter gene having a PAMP-inducible promoter (*pFRK1-Luc*) in tomato cells. Out of eight, three SFIs, SFI5, SFI6, and SFI7 were found interrupting MAPK signaling at or upstream of the MAPK cascade in tomato (host of *P. infestans*), but not in *Arabidopsis* (non-host of *P. infestans*). SFI3 was found to suppress reported gene activity downstream of MAP kinase activation, which is defined as post-translational MAP Kinase modification by phosphorylation, in tomato only (Zheng et al., 2014). Whereas SFI1, SFI2, and SFI8/Avrblb2 were found functionally redundant in both tomato and *Arabidopsis* as they suppressed transcriptional activation of flg22-induced marker genes. Interestingly, none of the 8 SFIs attenuated flg22-dependent post-translational MAP kinase activation in *Arabidopsis*. Thus, SFI1, SFI2 and SFI8 putatively act downstream of post-translational MAP kinase activation (Zheng et al., 2014). In a recent study, SFI3 was shown to be localized in the host's nucleus and interacted with a protein UBK that contains both U-box and kinase domains. Importantly, this SFI3-UBK interaction is found responsible for flg22-induced transcription responses (He et al., 2019). A *P. sojae* RXLR effector, Avh331 is reported to suppress INF1 induced PTI by significantly suppressing transcriptional activation of resistance marker genes downstream of the MAPK signaling, thus leading to decreased H_2_O_2_ accumulation and callose deposition to promote *Phytophthora* infection of *Arabidopsis* and *N. benthamiana* (Cheng et al., 2012). Another *P. sojae* effector, PsCRN63 (crinkler) is reported to suppress flg22 induced PTI through MAPK signaling (Li et al., 2016).

SFI8/Avrblb2 is a core *P. infestans* RXLR effector, found to have multiple host targets associated with diverse defense mechanisms. In addition to targeting MAPK signaling, SFI8/Avrblb2 and its other homologs interact with diverse calmodulins (CaMs) (Naveed et al., 2019). CaMs are ubiquitous Ca^2+^sensors in eukaryotes that play a major role in calcium signaling by interacting with numerous downstream targets. We have found that CaM binding to Avrblb2 is required for its recognition by the cognate R protein, Rpi-blb2 (Naveed et al., 2019). Like SFI8/Avrblb2, SFI5 is also shown to interact with CaMs to suppress PTI (Zheng et al., 2018). CaM binding to SFI5 was found essential for MAPK suppression (Zheng et al., 2018). Targeting of both MAPK and calcium signaling components by Avrblb2 and SFI5 indicates highly complex cross talk between these signaling pathways during all PTI, ETS and ETI responses (Oh et al., 2014b).

#### Modulation of Defense Hormones Signaling

As discussed above, plant hormone signaling plays a crucial role in cell death and SAR responses. It is generalized that SA mediates resistance to biotrophic pathogens, whereas JA and ET signaling has been associated with resistance to necrotrophs. Since most of the *Phytophthora* pathogens are hemibiotrophs, SA, JA, and ET were all found involved in contriving defense against *Phytophthora* pathogens (Halim et al., 2009; Kawamura et al., 2009; Savchenko et al., 2010; Chang et al., 2015).

Recently, Yang et al. reported ET biosynthesis pathway as an essential defense component against *P. sojae* infection in soybean. To counter ET mediated defense, *P. sojae* deploys PsAvh238, a RXLR effector that can interact with host's ACSs (aminocyclopropane carboxylic acid synthases). ACSs contribute to plant defense by catalyzing a critical step in the ET biosynthesis pathway. PsAvh238 is shown to destabilize ACSs to suppress ET production by the host and thus, promote infection (Yang et al., 2019). Two *P. capsici* RXLR effectors were shown to target different components of SA signaling. Enhanced Disease Susceptibility 1 (EDS1) was first discovered as *eds1* mutation in *Arabidopsis* that enhanced disease susceptibility against a biotrophic oomycete pathogen *Peronospora parasitica*. Further research revealed EDS1 as a positive regulator of plant immunity that interacts with Phytoalexin Deficient 4 (PAD4) and this EDS1-PAD4 complex plays a key role in SA accumulation and also regulate SA mediated defense (Wiermer et al., 2005). PcAvh103 is reported to promote *P. capsici* infection by binding with EDS1 and disrupting EDS1-PAD4 (Li et al., 2020). EDS1-PAD4 operates upstream of the SA pathway, whereas NPR1 (non-expressor of pathogenesis related1) is a SA receptor that operates downstream signaling. RxLR48 is found to associate with NPR1 to facilitate its nuclear localization and inhibits its proteasome-mediated degradation to suppress SA mediated defense and promote *P. capsici* infection (Li Q. et al., 2019b).

### RXLR Effectors Target Negative Defense Regulators Involved in Diverse Mechanisms

In addition to the above-mentioned examples, in which positive regulators of plant immunity are modulated by *Phytophthora* spp. effectors, there are numerous examples in which negative regulators of plant immunity are also affected by *Phytophthora* spp. effectors. These negative immune regulators are termed as susceptibility factors (S-factors). The best studied S-factor with practical application in agriculture is the barley *MLO* gene and its homologs in diverse plants. Plants harboring dominant MLO alleles are highly susceptible to powdery mildew pathogens, whereas plants carrying recessive *mlo* alleles are immune (Bai et al., 2008). Similarly, there are numerous other examples of diverse S-factors. Some *Phytophthora* effectors targeting the host S-factors to promote ETS have been discovered (Boevink et al., 2016a). *P. infestans* has been shown to promote AS of two S-factors, a receptor like kinase (RLPK) and a bHLH transcription factor (bHLH025), to promote infection (Huang et al., 2020). Some splicing regulatory effectors (SREs) reported in this study were previously shown to interact with host's S-factors. SRE2 (Pi04089) was reported to interact with K-homology RNA-binding protein (KRBP1), which is an S-factor because its overexpression significantly increased *P. infestans* colonization (Wang et al., 2015).

As is described above, ubiquitin mediated protein degradation is an important component of plant immunity and thus, is targeted by multiple RXLR effectors. Unlike, above mentioned examples of targeting positive immune regulators, Pi02860 (SRE1) targets an S-factor involved in host's protein degradation machinery. NRL1 (non-phototrophic hypocotyl 3/root phototropism 2 (NPH3/RPT2)-like protein), which encodes a substrate adaptor component of a CULLIN3-associated ubiquitin E3 ligase in plants (He et al., 2018), is reported as S-factor because it favors the colonization of *P. infestans* in potato (Yang et al., 2016). A potential substrate of NRL1, SWAP70 (a guanine nucleotide exchange factor), was found as a positive regulator of immunity against *P. infestans* because it promotes INF1-mediated PTI. SRE1 was reported to attenuate this INF1-induced PTI responses through its interaction with NRL1 to enhance proteasome-mediated degradation of StSWAP70 (He et al., 2018).

In a recent report, a *P. capsici* effector, RxLR207, is shown to promote the 26S proteasome-dependent degradation of multiple proteins involved in ROS homeostasis (Li Q. et al., 2019a). RxLR207 is found to interact with BPA1 (binding partner of ACD11 [Accelerated cell Death11]) and four other members of BPLs (BPA1-Like proteins). ACD11 and BPAs are negative regulators of cell death as mutations in these genes show constitutive cell death. Since *P. capsici* and other *Phytophthora* spp. transition from a biotrophic to necrotrophic phase, in which plant cells and tissues die, and since RxLR207 is involved in this transition (Li Q. et al., 2019a), it can be speculated that RxLR207 promotes cell death by destabilizing BPAs. Unlike the above examples of RXLR promoted protein degradations, *P. sojae* effector PsAvh262 is reported to stabilize ER luminal binding immunoglobulin proteins (BiPs), as they are shown to act as S-factors because they suppress host's ER stress-triggered cell death and thus promote *Phytophthora* infection (Jing et al., 2016), perhaps at the biotrophic phase.

Another RXLR effector of *P. infestans*, Pi17316 is shown to target a MAP3K, a vascular highway1-interacting kinase (StVIK) to suppress INF1 induced PTI (Murphy et al., 2018). The interaction of Pi17316-StVIK (MAP3K) is different from other effector-MAPK interaction in a way that StVIK is a S-factor. Enhanced expression of both Pi17316 and StVIK is shown to suppress INF1 induced defense and promote *P. infestans* colonization. Silencing of NbVIK attenuates the ability of Pi17316 to suppress INF1 induced cell death and to promote disease. These results clearly showed the potential of StVIK and NbVIK as S-factors that promote late blight disease (Murphy et al., 2018).

Recently, Avr3a like effectors of three *Phytophthora* spp. were reported to interact with specific cinnamyl alcohol dehydrogenases (CADs) from *Arabidopsis* (AtCAD7) and *N. benthamiana* (NbCAD7). CADs are reported to contribute to structural lignification during plant cell development and some of them are specifically induced upon pathogen invasion to increase localized lignification that acts as a barrier against infection. However, both AtCAD7 and NbCAD7 are reported as S-factors against *Phytophthora* infection as their silencing resulted in reduced host susceptibility. Moreover, NbCAD7 was found to amplify the suppression of INF1 induced cell death by PiAvr3a and AtCAD7 suppressed core immune responses like ROS burst, callose deposition and WRKY33 expression (Li T. et al., 2019).

### RXLR Effectors Target S-Factors and Positive Defense Regulators to Modulate Growth Hormone Signaling

Extensive research in diverse plant-pathogen systems, including *Phytophthora*, reveals a complex crosstalk—exhibiting both complementary and antagonizing effects- among growth- and defense-related phytohormone signaling (Turnbull et al., 2019). RXLR effectors have also been found to interfere with plant growth hormone signaling by targeting different signaling components that play a positive or negative role in host defense.

PcAvh1, a *P. capsici* RXLR effector, targets the scaffolding subunit of the protein phosphatase 2A (PP2Aa) involved in positively regulating plant immunity and growth. PP2A is involved in auxin signaling and silencing of PP2Aa in *N. benthamiana* resulted in attenuation of resistance to *P. capsici* as well as dwarfism of the plant (Michniewicz et al., 2007; Chen et al., 2019). The *P. infestans* RXLR effector, PiAvr2, has been shown to highly induce brassinosteroid (BR) hormone signaling. The BRI1-SUPPRESSOR1-like (BSL1) potato phosphatases are the components of BR signaling pathway crucial for plant growth. PiAvr2 was found to interact with all three BSL family members, StBSL1, StBSL2, and StBSL3 to attenuate immunity and enhance host susceptibility to *P. infestans*. Both BSL1 and BSL3 are shown to suppress INF1 induced cell death, whereas BSL2 and BSL3 are required for BSL1 stability. Moreover, PiAvr2 also caused the constitutive overexpression of bHLH transcription factor StCHL1, which is known to regulate antagonism between growth and immunity. It has also been found that suppression of INF1 induced cell death by StBSL1 and StBSL3 requires StCHL1. Since all these BR signaling components, BSLs and the TF CHL1 are negative regulators of host immunity, they all serve as S-factors (Turnbull et al., 2017, 2019). Another RXLR effector, Pi04314 has been shown to enhance leaf colonization of *P. infestans* by modulating JA and SA signaling (Boevink et al., 2016b). Pi04314 translocates to the host nucleus where it interacts with three isoforms of PP1c (protein phosphatase 1 catalytic) proteins to re-localize them from nucleolus to nulcleoplasm and also attenuate the induction of JA and SA responsive genes. PP1cs serve as S-factors because Pi04314-PPIcs interaction has shown to increase host susceptibility to *P. infestans* (Boevink et al., 2016b). As PiAvr2 acts to suppress INF1 induced immunity by switching host hormonal machinery toward growth enhancement and Pi04314 do so by suppressing the host defense (other than INF1 induced) related hormone signaling, it can be concluded that *P. infestans* is capable of differentially manipulating different types of hormone signaling pathways to attenuate PTI induced by different elicitors to promote late blight disease.

## Effector Triggered Immunity (ETI) and ETS2

Effectors work as a double edge sword, which on one side suppress host defense responses, but on the other side act as avirulence (Avr) factors inducing R protein-mediated defenses in plants (Figure 3). This dual capability of effectors is well-represented in the ETS and ETI phases of the zigzag model (Jones and Dangl, 2006). All known *Phytophthora R* genes in potatoes are activated by effectors from *Phytophthora* spp. Introgression of *R* genes effective against *Phytophthora* spp. from wild Solanaceous plants into cultivated crop plants is a viable method against late blight disease, and more than a dozen *R* genes effective against various *Phytophthora* spp. have been identified. However, deployment of these single genes does not provide stable resistance against the rapidly evolving populations of *P. infestans* (Armstrong et al., 2005; Lokossou et al., 2009). *R1, R3a*, and *R4* are among the 11 major dominant *R* genes introgressed from *Solanum demissum* into different potato cultivars. R1 recognizes *P. infestans* RXLR effector Avr1 to induce ETI. As mentioned above, Avr1 circumvents PTI by suppressing callose deposition (Du Y. et al., 2015). *P. infestans* isolates virulent on R1 containing potato plants lack Avr1 but carry a homologous gene named Avr1-like (Avr1-L) effector, which, due to a deletion of potent domains at the C terminus, is not recognized by R1 (Du et al., 2018). Similarly, *P. infestans* isolates virulent on potato cultivars carrying R4 gene carry truncated and non-functional cognate avr4 alleles (Van Poppel et al., 2008). The *P. infestans* RXLR effector, Avr3a is recognized by R3a. Interestingly, two *P. sojae* RXLR effectors, Avr1b and Avh1b have sequence similarity to Avr3a but only Avh1b is found to be weakly recognized by R3a whereas Avr1b like virulent alleles of Avr3a evade recognition by potato R3a. Avr1b is recognized by a soybean R protein Rpi1b and virulent isolates avoid recognition by either suppressing the expression of functional Avr1b or carry allele having multiple amino acid substitutions (Armstrong et al., 2005).

According to the zigzag model, perception of Avr effectors by the cognate R proteins can be either through their direct physical interaction or indirectly through recognizing modifications in host proteins targeted by these effectors. In case of *Phytophthora-*plant interactions, there is only one evidence of direct physical interaction-based recognition of class I variants of IPI-O (e.g., IPI-O1/Avrblb1, IPI-O2) family of *P. infestans* RXLR effectors by Rpi-blb1 that is a coiled-coil type NB-LRR (CC-NB-LRR) type R protein. Whereas class III variants of IPI-O family (i.e., IPI-O4) have also been found to physically bind with Rpi-blb1 but this interaction doesn't lead to induction of defense responses. Binding of IPI-O4 with Rpi-blb1 blocks recognition of IPI-O1 and also leads to the suppression of IPI-O1 elicited HR response (Chen et al., 2012). *P. infestans* lineages carrying IPI-O4 cause more severe disease in hosts harboring Rpi-blb1 and recently, evidence of suppression of Rpi-blb1 associated resistance is provided by *in planta* over-expression of IPI-O4 (Chen and Halterman, 2017). This IPI-O4 mediated suppression of IPI-O1 and Rpi-blb1 mediated ETI align well with the ETS2 phase of zigzag model.

Another CC-NB-LRR type *R* gene identified from *S. bulbocastanum* is *Rpi-blb2* that can recognize PiAvrblb2, originally discovered as *Avr* factor. Transformation of this *R* gene into potato conferred broad-spectrum resistance against all the then known races of *P. infestans* (Song et al., 2003). *Rpi-blb2* is one of the most well-studied *R* gene in terms of *Phytophthora-*plant interactions. The detailed mechanism behind this resistance is not fully explored but it seems to be involved in calcium and SA signaling in Rpi-blb2-mediated ETI. Transgenic *N. benthamiana* harboring *Rpi-blb2* gene displayed strong HR in response to transient expression of the PiAvrblb2, through SGT1 and SA mediated pathways (Oh et al., 2014a,b). SGT1 is a eukaryotic ubiquitin ligase-associated co-chaperone that is widely reported to be involved in both PTI and ETI associated HR responses (Austin et al., 2002; Li et al., 2016). The *P. capsici* and *P. infestans* INF1 (PAMPs) and PexRD2 (RXLR effector) induced-HR is also shown to require SGT1 (Oh et al., 2009; Liu et al., 2016). Cross talk between SGT1 and calcium signaling pathways has been reported to regulate immune responses (Nowotny et al., 2003; Liu et al., 2016). In our laboratory, we showed that physical interaction of Avrblb2 with calmodulin is required for its recognition by Rpi-blb2 (Naveed et al., 2019). Based upon all these evidences, we suggest that calmodulin serves as a guardee, which is monitored by Rpi-blb2 to induce resistance response.

Other classes of four *P. infestans* resistant R proteins, Rpi-blb3, Rpi-abpt, R2, and R2-like harboring a characteristic leucine zipper nucleotide binding site leucine-rich repeat (LZ-NB-LRR) domain were cloned from different wild potato species. All these *Rpi* genes recognize PiAvr2 and trigger strong HR response (Lokossou et al., 2009).

Avr2 is a multiallelic highly divergent class of RXLR effectors that are recognized by the NB-LRR type R protein, R2 (Yang et al., 2020). As described above, PiAvr2 promotes ETS by modulating BR hormone signaling. Interestingly, both virulent and avirulent Avr2 alleles interact with the host phosphatase BSL1 but only the avirulent variants mediate activation of R2 (Saunders et al., 2012). This and additional studies suggest that BSL family proteins act as guardees and contribute toAvr2-mediated virulence of *P. infestans* (Turnbull et al., 2019). Many different R2 alleles and orthologs from six diverse *Solanum* spp. have been reported to recognize different Avr2 variants. *P. infestans* strains, virulent on R2 containing potatoes were found to either suppress Avr2 expression or carry a distinct variant of Avr2 that eludes R2 mediated perception (Gilroy et al., 2011a). Recently, Yang et al. (2020) discovered that virulent and avirulent Avr2 variants have different protein structures and virulent Avr2 are in fact intrinsically disordered proteins (IDPs). IDPs carry intrinsically disordered regions (IDRs) that may endow these proteins to have functional and evolutionary advantages over structured proteins. IDP- type Avr2 variants are predicted to be unstable and have a short protein half-life. The authors have suggested that these properties enable virulent forms of Avr2 effectors to elude recognition by R2 (Yang et al., 2020). This is an excellent example of effector evolution to circumvent ETI and cause ETS.

All these examples indicate that *Phytophthora* spp. carry multiple rapidly evolving effectors that aid them to overcome ETI and enter into the second phase of ETS. On the basis of extensive genome analysis of different *Phytophthora* spp. Haas et al. (2009) reported extensive sequence diversity among both inter and intraspecies RXLR and CRN effectors with high degree of expansion and pseudogene formation. Both RXLRs and CRNs are modular proteins with highly conserved N terminal domains and highly diverse C termini. Moreover, these effector genes typically reside in the gene sparse and repeat-rich genomic environment. Mobile elements in these repetitive regions contribute to the dynamic nature and high rates of gene gain and gene loss observed for these effectors (Haas et al., 2009). Although, both CRNs and RXLRs are shown to have the potential to contribute to the plant-pathogen evolutionary arm's race, currently, all known effectors with avirulence functions and cognate *R* genes belong to RXLR effectors.

## Perspective on Developing *Phytophthora*-Resistant Plants

As discussed above, plant defense against *Phytophthora* spp. is a complex multilayered, interlinked phenomenon where each defense layer is intervened by effectors that have been rapidly coevolving during host-*Phytophthora* interactions. In order to develop sustainable resistance in crop plants against these noxious pathogens, a thorough understanding of the molecular basis of *Phytophthora-*plant interactions and discovery of multiple sources of genetic resistance are very important. In the past, several *P. infestans R* genes have been identified in wild potato, tomato, and other plants and are being used to develop *Phytophthora-*resistant plants (Zhu et al., 2012; Rodewald and Trognitz, 2013). But resistance was broken down because most of these *R* genes are very specific in recognizing their *Phytophthora* counterparts (*avr* genes) that are diverse not only among different *Phytophthora* spp. but also among different races and strains of the same species (Rodewald and Trognitz, 2013). Thus, the *R* gene-mediated resistance is not sufficient alone to provide broad-spectrum and durable resistance.

Apart from this effector-*R* gene model, recent discoveries of host's S-factors being targeted by multiple RXLR effectors is opening up new horizons for developing *Phytophthora* resistant crops. Genome editing through CRISPR/Cas-9 technology provides an excellent tool to knock out the negative immune regulators in plants. Efforts to implement these latest developments have already started and are showing promising results. For example, silencing of six S-genes in potato resulted in enhanced resistance against *P*. *infestans* (Sun et al., 2016).

In this era of high throughput technology, *Phytophthora*-plant interactions are being widely studied using different “omics” (genomics, transcriptomics, proteomics, metabolomics, and effectromics) approaches to identify novel components of plant defense. For example, comparative transcriptome studies of *P*. *infestans*-resistant wild tomato enabled the identification of *P*. *infestans* resistant transcription factors SpWRKY3 (Cui et al., 2018), and a long non-coding RNA (lncRNA16397) along with glutaredoxin (SpGRX) gene. Plants transformed with these non-race specific genes conferred broad spectrum resistance against *P. infestans* (Cui et al., 2017).

PAMP initiated non-host resistance have also been explored widely in an effort to identify non-race-specific sources of resistance. Recently, ELR that can recognize elicitins from diverse *Phytophthora* spp. was identified from a wild potato species *S. microdontum*. Transfer of ELR in cultivated potatoes exhibit enhanced resistance to diverse strains of *P. infestans* (Du J. et al., 2015). Identification of such non-race-specific sources of resistance and their pyramiding with multiple *R* genes and S-factors can offer stable broad-spectrum resistance against these rapidly evolving *Phytophthora* spp.

## Summary and Conclusion

Most of the findings about *Phytophthora-*plant interactions described above are well-aligned with the basic zigzag model. However, there are some conflicts among the basic designation of components and the defense responses that contribute in PTI and ETI (Thomma et al., 2011). Based on wide distribution among diverse pathogen groups, and contribution in pathogen fitness and virulence as well as evasion of plant defense in multiple ways, some apoplastic effectors fulfill the criteria to be designated as PAMPs as well as effectors. In the zigzag model, the term *R* gene is confined to intracellular NB-LRR domain containing receptors that recognize effectors in the cytoplasm of the host cells to initiate quick defense responses up to a level where the threshold to trigger HR and SAR is attained (Jones and Dangl, 2006). Both known PRRs for *Phytophthora* spp. PAMPs, ELR, and RLP23 belong to RLPs that are currently considered a subclass of R proteins. Moreover, the timing, magnitude and the outcome of the defense responses cannot be strictly restricted to either PTI or ETI (Thomma et al., 2011). For example, as described above almost all *Phytophthora* spp. PAMPs have been found to induce HR and SAR upon their recognition and also the defense responses induced by CRN effectors have been found to develop slowly as compared to INF1 induced responses (Torto et al., 2003).

As mentioned above *Phytophthora* spp. PAMPs belong to diverse classes of biological molecules i.e., carbohydrates, proteins, and lipids. The pathogenic and evolutionary success of *Phytophthora* spp. can be ascribed to their diverse and rapidly evolving effector gene complements. Particularly, the RXLR effectors target diverse host proteins involved in different cellular processes to intervene host defense in multiple ways. Broadly, RXLR effectors can either suppress the positive regulators or support the negative regulators of plant defense to establish infection. Although evidence of defense modulation through intervention of diverse signaling pathways and physiological mechanisms have been found, compared to the 100s of *Phytophthora* effectors, these examples are just the tip of an iceberg. Moreover, unlike PAMPs, effector biology research is mainly limited to two pathosystems; *P. sojae*-soybean and *P. infestans*-tomato/potato/tobacco. In order to thoroughly understand the *Phytophthora-*plant interactions, there is dire need to expand the research beyond these model pathosystems.

## Author Contributions

ZN, GA, and JC conceived the idea. ZN designed the layout of the article and wrote the paper. ZN and HM contributed to the figures. XW and GA revised and approved the final version of the manuscript. All authors contributed to the article and approved the submitted version.

## Conflict of Interest

GA was employed by EukaryoTech LLC. The remaining authors declare that the research was conducted in the absence of any commercial or financial relationships that could be construed as a potential conflict of interest.
